# The Association Between Obesity and Risk of Acute Kidney Injury After Cardiac Surgery

**DOI:** 10.3389/fendo.2020.534294

**Published:** 2020-10-06

**Authors:** Ning Shi, Kang Liu, Yuanming Fan, Lulu Yang, Song Zhang, Xu Li, Hanzhang Wu, Meiyuan Li, Huijuan Mao, Xueqiang Xu, Shi-Ping Ma, Pingxi Xiao, Shujun Jiang

**Affiliations:** ^1^Clinical Metabolomics Center, China Pharmaceutical University, Nanjing, China; ^2^State Key Laboratory of Natural Medicines, China Pharmaceutical University, Nanjing, China; ^3^Department of Nephrology, Jiangsu Province Hospital, Nanjing, China; ^4^Department of Cardiology, The Affiliated Sir Run Run Hospital of Nanjing Medical University, Nanjing, China; ^5^Department of Infectious Diseases, Nanjing Hospital of Chinese Medicine Affiliated to Nanjing University of Chinese Medicine, Nanjing, China

**Keywords:** obesity, body mass index, acute kidney injury, cardiac surgery, creatinine

## Abstract

**Objective:**

To determine the relationship between obesity and the risk of AKI after cardiac surgery (CS-AKI) in a cohort study.

**Methods:**

A total of 1,601 patients undergoing cardiac surgery were collected and their incidence of CS-AKI was recorded. They were divided into underweight, normal weight, overweight, and obese groups. Logistic regression was used to estimate the association between BMI (body mass index) and CS-AKI risk. Then, a meta-analysis of published cohort studies was conducted to confirm this result using PubMed and Embase databases.

**Results:**

A significant association was observed in this independent cohort after adjusting age, gender, hypertension and New York Heart Association classification (NYHA) class. Compared with normal BMI group (18.5 ≤ BMI < 24.0), the individuals with aberrant BMI level had an increased AKI risk (OR: 1.68, 95% CI: 1.01–2.78) for BMI < 18.5 group and (OR: 1.43, 95% CI: 0.96–2.15) for BMI ≥ 28.0. Interestingly, the U-shape curve showed the CS-AKI risk reduced with the increasing of BMI when BMI ≤ 24.0. As BMI increases with BMI > 24.0, the risk of developing CS-AKI increased significantly. In the confirmed meta-analysis, compared with normal weight, overweight group with cardiac surgery had higher AKI risk (OR: 1.28, 95% CI: 1.16–1.41, *P*_heterogeneity_ = 0.49). The similar association was found in obesity subgroup (OR: 1.79, 95% CI: 1.57–2.03, *P*_heterogeneity_ = 0.42).

**Conclusion:**

In conclusion, the results suggested that abnormal BMI was a risk factor for CS-AKI independently.

## Introduction

Acute kidney injury (AKI) is a multifactorial, complicated, heterogeneous clinical syndrome characterized by a sudden decrease of renal function. It is considered to be one of the most common serious complications after cardiac surgery (1), which not only prolongs hospitalization, but also increases the risk of death (2–5). About 18% of patients undergoing cardiac surgery develop AKI, and between 2% and 6% need renal replacement therapy (6). More than 2 million patients worldwide undergo cardiac surgery annually, while the incidence of post-cardiac surgery AKI (CS-AKI) varies from 5% to 42% (7, 8). CS-AKI results from reduced renal perfusion which in turn results from post-cardiac surgery vasoconstriction or ischemia–reperfusion injury that occurs in the extracorporeal circulation (9).

Obesity is an increasingly relevant medical and socio-economic problem in developed and developing countries (10). According to the World Health Organization (11), adults with body mass index (BMI) ≥ 25.0 kg/m^2^ and ≥ 30.0 kg/m^2^ are defined as overweight and obese, respectively. Cardiac patients with excessive BMI are at an increased risk of AKI, owing to their disproportionately higher burden of comorbidities and underlying structural changes that occur in the kidneys of obese patients in spite of normal serum chemistry (12). Obesity is reported to be associated with AKI for patients under intensive care, post-operative populations (13–15) and obese patients after surgery with AKI exhibited the increase of oxidative stress, endothelial dysfunction and inflammation (16, 17). However, another study found no correlation between obesity and increased risk of major perioperative complications after transcatheter aortic valve implantation (18). Thus, the association between CS-AKI and obesity remains questionable.

Herein, we recruited CS-AKI patients and performed a prospective cohort study to determine the association between obesity and CS-AKI. Thereafter, a meta-analysis was conducted to confirm or otherwise our findings.

## Methods And Material

### Clinical Study Population

Blood samples from participants undergoing cardiac surgery were collected at the Jiangsu Province hospital. The BMI classification followed the standards established for Chinese by the Department of Disease Control, Ministry of Health (19). Patients (n = 1,601) were divided into four groups: underweight (BMI < 18.5, n = 98); normal-weight (18.5 ≤ BMI < 24.0, n = 799); overweight (24.0 ≤ BMI < 28.0, n = 539); and obese (BMI ≥ 28.0, n = 165) groups. AKI was defined in accordance with the guidance of the Kidney Disease Improving Global Outcomes (KDIGO) (19), thus: (1) increase in serum creatinine by ≥0.3 mg/dl (≥26.5 μmol/L) within 48 h; (2) increase in serum creatinine to ≥1.5 times baseline, which is known or presumed to have occurred within previous 7 days; (3) urine volume <0.5 ml/kg/h for 6 h and staged according to the serum creatinine and urine output. The preoperative general information of all patients was collected. A detailed clinical history was obtained from the patient and the family, including gender, age, smoking history, history of disease (hypertension, diabetes, hyperlipidemia). Blood biochemical indices were measured with Biochemical Analyzer (BECKMAN COULTER AU5800, Japan), in which the creatinine was detected by the enzymatic method with creatininase coupled sarcosine oxidase method. All medical laboratory data were generated by the clinical laboratory of Jiangsu Province hospital.

All participants provided written informed consent at the time of enrollment. The inclusion/exclusion criteria are described as following, for inclusion criteria: (1) Patients who have undergone cardiac surgery; (2) AKI was determined by in-hospital examination; (3) Informed consent was signed. For exclusion criteria: (1) Patients with elevated creatinine before operation or those were diagnosed as severe renal insufficiency before operation; (2) Patients with chronic kidney disease, kidney surgery or kidney transplantation; (3) Patients with malignant tumor, autoimmune disease, severe infection and trauma. This study complied with the Helsinki Declaration.

The perioperative standard management, coronary angiography, anesthesia and surgical techniques were performed in all patients, and the operation and cardiopulmonary bypass were performed by the same professional team. American society of anesthesiologist classification, blood gas analysis, intraoperative medication, intraoperative fluid output, operation time, cardiopulmonary bypass time, aortic block time and cardiac arrest time were monitored during surgery.

All patients were transferred to the intensive care unit (ICU) after operation, and laboratory indicators will be standard monitored all the time. After 24-48 hours of routine treatment, the patients can be transferred to the cardio vascular ward. All enrolled patients avoided the use of potentially nephrotoxic drugs during hospitalization.

### Search Strategy and Selection Criteria

Studies published up to 1 December 2018 were searched for in PubMed and Embase databases using the following key words: (acute kidney injury) AND (obesity), (acute kidney injury) AND (BMI). All titles and abstracts of studies retrieved were reviewed to select potentially eligible studies. Full texts of potentially eligible publications were reviewed by all investigators. Discrepancies were reconciled through discussion by the reviewers who extracted the data and, if these remained unresolved, the other authors were involved in arriving at a resolution. Our outcome was limited to CS-AKI by any of these definitions. The references of the articles that satisfied the inclusion criteria were also involved, and duplicated studies were excluded.

The inclusion criteria are described here: (1) studies confirmed to be cohort; (2) studies that set BMI, obesity or weight the exposure factor(s); (3) articles describing the occurrence of CS-AKI; (4) studies that provided the value of events with Odds Ratio (OR) and 95% Confidence Interval (CI).

### Data Extraction

We extracted the following information from each study: first author’s surname, publication year, study location, study period, sample size, sample source, AKI diagnostic criteria, BMI, BMI categories, risk estimates with the corresponding 95% CIs for each BMI category, and adjustment factors in the multivariable analysis. The BMI (kg/m^2^) for adults of European and American decent was classified as follows: normal weight, 18.5 or 20.0–25.0; overweight, 25.0–30.0 and obesity ≥ 30.0 (using the standards of the World Health Organization). One study from China used BMI classification established for Chinese, hence, underweight < 18.5; normal-weight 18.5–24.0; overweight 24.0–28.0; obesity BMI ≥ 28.0 (20). We assigned the midpoint of the upper and lower boundaries in each category as the average level. Data were extracted independently according to the selection criteria.

### Statistical Analysis

Categorical data were shown as percentage, while continuous data were provided as mean ± SD. The normal weight group was regarded as reference category in the study. Cohort logistic regression was used to estimate the association between BMI and CS-AKI, and odd ratio and 95% CI were reported. We adjusted for covariates that have been used in previous studies for prediction of CS-AKI, including patient demographics (age, sex) and medical history (hypertension, NYHA class). Categorical variables were compared with chi-square test while predictors of CS-AKI were identified with logistic regression analysis.

For the meta-analysis, combined OR was used to measure the association between obesity and CS-AKI risk. Stratification of meta-analysis was conducted for different levels of BMI. Data were analyzed using a random-effects model. To investigate the effect of potential confounders, subgroup analyses were conducted using the available characteristics of studies and participants, if three or more studies were available per subgroup.

The *Q* and *I*^2^ statistics were used to assess heterogeneity among studies. To explore the possible heterogeneity among different studies, AKI diagnostic criteria, study locations, and adjustments for confounding factors, a meta-regression model was used. Considering the possibility of effect modification by other known risk factors (i.e., sex, age, smoking, and hypertension), we also conducted dose-response meta-analyses by these factors respectively apart from subgroup analyses. Sensitivity analysis was used to evaluate the influence of a single study on the entire result by omitting each study in sequence. Publication bias was evaluated with Begg’s funnel plots and the Egger’s test.

All statistical analyses of clinical information were performed with R 3.5.1. All tests were two-sided and *p* < 0.05 was considered statistically significant.

## Results

### Patients’ Characteristics

A total of 1,601 cardiac surgery patients were recruited from the Jiangsu Province hospital for this study. Baseline characteristics and laboratory data are shown in **Table 1**. In detail, there were 318 AKI patients and 1,283 controls. Age, gender, comorbid conditions (diabetes, hypertension, and hyperlipemia), previous surgical history, smoking, New York Heart Association classification (NYHA) class, and coronary angiography (CAG) are provided.

**Table 1:** Baseline characteristics of subjects.

	AKI (n = 318)	Non-AKI (n = 1,283)	*P* value
**Male (%)**	187(58.91)	649(50.58)	0.009
**Age (mean ± SD)**	56.92 ± 11.40	53.57 ± 13.58	0.001
**BMI (%)**			
**BMI < 18.5 (n = 98)**	24(7.55)	74(5.77)	0.658
**18.5 ≤ BMI<24 (n = 799)**	150(47.17)	649(50.58)	0.229
**24 ≤ BMI<28 (n = 539)**	103(32.39)	436(33.98)	0.298
**BMI ≥ 28 (n = 165)**	41(12.89)	124(9.66)	0.045
**Medical history**			
**Diabetes (%)**	52(16.35)	170(13.25)	0.152
**Hypertension (%)**	146(45.91)	433(33.75)	<0.001
**Hyperlipemia (%)**	80(25.16)	268(20.89)	0.099
**Previous Surgery history (%)**	145(45.60)	531(41.39)	0.174
**Smoking (%)**	90(28.30)	303(23.62)	0.082
**NYHA class (%)**			
** Ⅰ**	29(9.12)	168(13.09)	0.053
** Ⅱ**	73(22.96)	424(33.05))	<0.001
** Ⅲ**	189(59.43)	612(47.70)	<0.001
** Ⅳ**	27(8.49)	79(6.16)	0.134
**CAG (%)**	141(44.34)	494(38.50)	0.057

BMI, body mass index; NYHA, New York Heart Association (classification); CAG: coronary arteriongraphy; AKI, acute kidney injury.

The percentage of males was higher among AKI patients than in non-AKI subjects. As for patients’ post-cardiac surgery, AKI group was older and exhibited higher percentage of hypertension and hyperlipemia than non-AKI patients. The NYHA class showed significant differences between AKI and non-AKI patients. In addition, more AKI subjects suffered from CAG than those in the non-AKI group.

With respect to BMI, there were 24 AKI patients in the underweight group (BMI < 18.5, n = 98), 150 AKI patients in the normal weight group (18.5 ≤ BMI < 24.0, n = 799), 103 patients in the overweight group (24.0 ≤ BMI < 28.0, n = 539), and 95 patients in the obese group (BMI ≥ 28.0, n = 95). The incidence of AKI was 24.49% for the underweight group, 18.77% for the normal weight group, 19.11% for the overweight group, and 24.85% for the obese group.

### The Association Between BMI and CS-AKI

**Figure 1:**
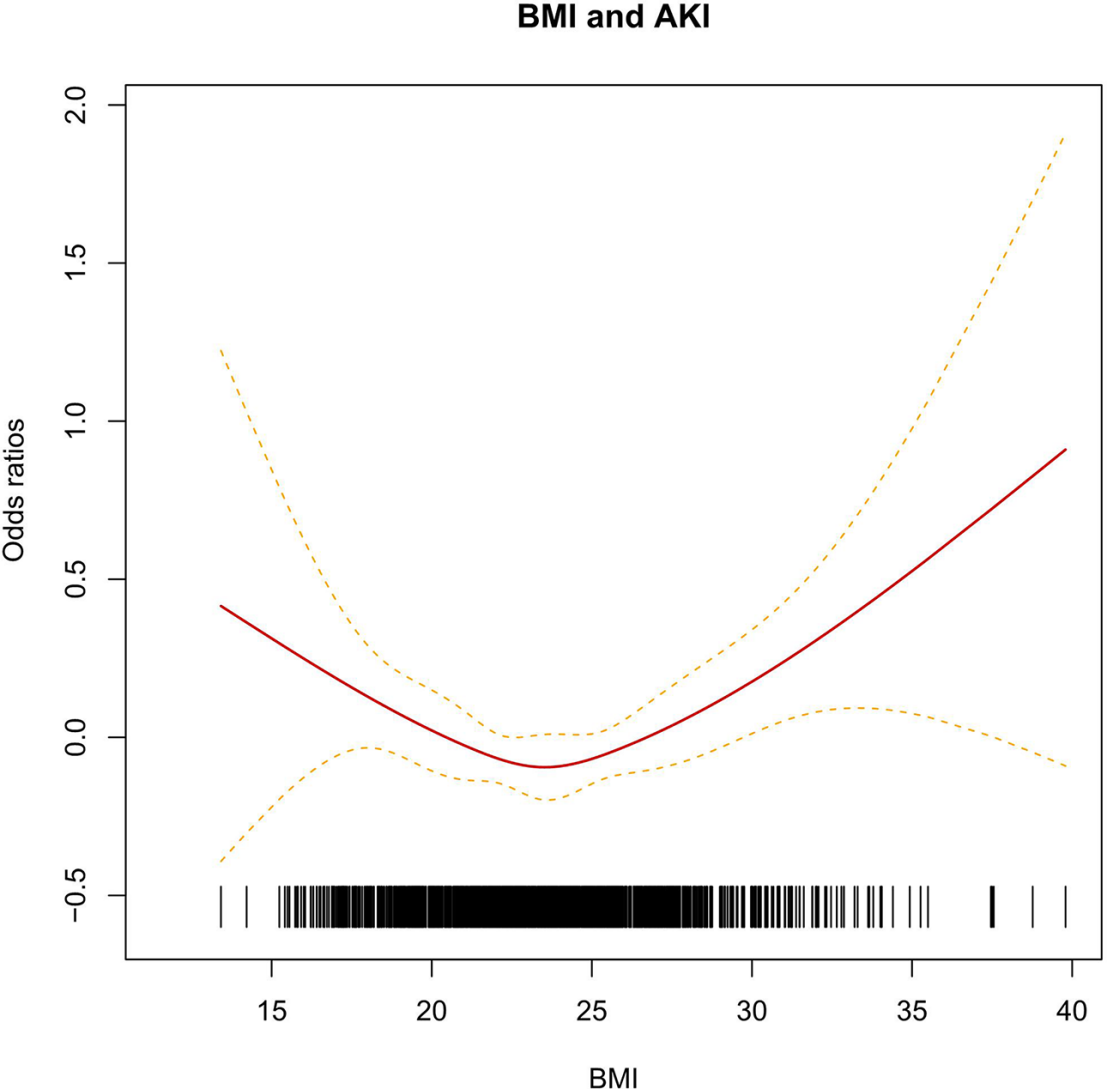
The linear relationship between BMI and AKI. The short black vertical line respect different BMI of individuals. The red solid line and the dash line respect the estimated relative risk and its 95% CI.

To estimate the relationship between BMI and CS-AKI risk, a logistic regression was conducted. A significant association was observed in this independent cohort after adjusting for age, gender, hypertension and NYHA class. As shown in **Table 2**, compared with normal BMI group (18.5 ≤ BMI < 24.0), the individuals with aberrant BMI level had an increased AKI risk (OR: 1.68, 95% CI: 1.01–2.78) for BMI < 18.5 group and (OR: 1.43, 95% CI: 0.96–2.15) for BMI > 28.0. Interestingly, the U-shape curve showed the CS-AKI risk reduced with increasing BMI (at BMI ≤ 24.00). As BMI increased beyond 24.0, the risk of developing CS-AKI increased significantly (**Figure 1**).

**Table 2:** The association between BMI and AKI.

Group	AKI	Non-AKI	OR (95%CI)	*P* value
**BMI < 18.50**	24	74	1.68 (1.01–2.78)	0.045
**18.50 ≤ BMI < 24.0**	150	649	1.00	1.00
**24.0 ≤ BMI < 28.0**	103	436	0.93 (0.70–1.24)	0.62
**BMI ≥ 28.0**	41	124	1.43 (0.96–2.15)	0.08

OR, Odds ratios represent BMI increases adjusted for age, gender, hypertension and NYHA class.

BMI, body mass index; NYHA, New York Heart Association (classification).

Stratified analyses performed according to BMI shown in **Table 3**, age, hypertension, and NYHA class were significantly associated with the risk of CS-AKI after adjusting age, gender, hypertension and NYHA class these factors (**Table 3**). The frequency of hypertension was positively associated with BMI increase.

**Table 3:** Subgroup analysis of clinical characteristics according to BMI.

	BMI<18.5 (n= 98)	18.5≤BMI<24 (n= 799)	24≤BMI<28 (n= 539)	BMI≥28 (n= 165)	All	*P* value
**Male (%)**	32 (32.65)	382 (47.81)	327 (60.67)	95 (57.58)	836 (52.22)	0.141
**Age (mean ±SD)**	46.69±16.37	53.00±13.73	57.00±11.61	55.00±11.48	54.00±13.24	0.030
**BMI (mean ±SD)**	17.22±0.95	21.61±1.50	25.68±1.14	30.35±2.20	23.61±13.56	0.001
**Medical history**						
**Diabetes (%)**	4 (4.08)	64 (8.01)	106 (19.67)	37 (22.42)	211 (13.18)	0.841
**Hypertension (%)**	15 (15.31)	45 (5.63)	207 (38.40)	86 (52.12)	353 (22.05)	0.008
**Hyperlipemia (%)**	14 (14.29)	145 (18.15)	134 (24.86)	55 (33.33)	348 (21.74)	0.962
**Previous surgery** **history (%)**	23 (23.47)	169 (21.15)	268 (49.72)	80 (48.48)	540 (33.73)	0.201
**Smoking (%)**	12 (12.24)	160 (20.03)	150 (27.83)	51 (30.91)	373 (23.30)	0.923
**NYHA class (%)**						<0.001
**Ⅰ**	13 (13.27)	87 (10.89)	99(18.37)	17 (10.30)	136 (8.49)	
**Ⅱ**	25 (25.51)	250 (31.29)	180 (33.40)	47 (28.48)	502 (31.36)	
**Ⅲ**	54 (55.10)	429 (53.69)	235 (43.60)	84 (50.91)	802 (50.09)	
**Ⅳ**	6 (6.12)	33 (4.13)	25 (4.64)	17 (10.20)	81 (5.06)	
**CAG (%)**	21 (21.43)	278 (34.79)	258 (47.87)	78 (47.27)	635 (39.66)	0.255
**AKI (%)**	24 (24.49)	150 (18.77)	103 (19.11)	41 (24.85)	318 (19.86)	0.147

BMI, body mass index; NYHA, New York Heart Association (classification); CAG: coronary arteriongraphy; AKI, acute kidney injury.

### Literature Search and Study Characteristics of Meta-Analysis

To further confirm the association between BMI and AKI risk, a meta-analysis was performed. A flowchart of this is shown in **Supplementary Figure 1**. The initial search of literature identified 768 unique studies, of which 705 were excluded. After carefully reviewing the full textual content of prospective studies, seven articles were finally used (18, 21–26). The characteristics of these studies are presented in **Supplementary Table 1**. In detail, three AKI diagnostic criteria were applied to these studies as following: three studies used AKIN criteria, two studies used RIFLE criteria, one study used KDIGO criteria and one study unknown. These studies were representative of four regions, namely USA, UK, Europe (including Italy, France, and The Netherlands) and China. The sample size ranged from 376 to 8,455, and the AKI cases ranged from 26 to 2,855.

### Quantitative Synthesis and Subgroup Analysis by Meta-Analysis

**Figure 2:**
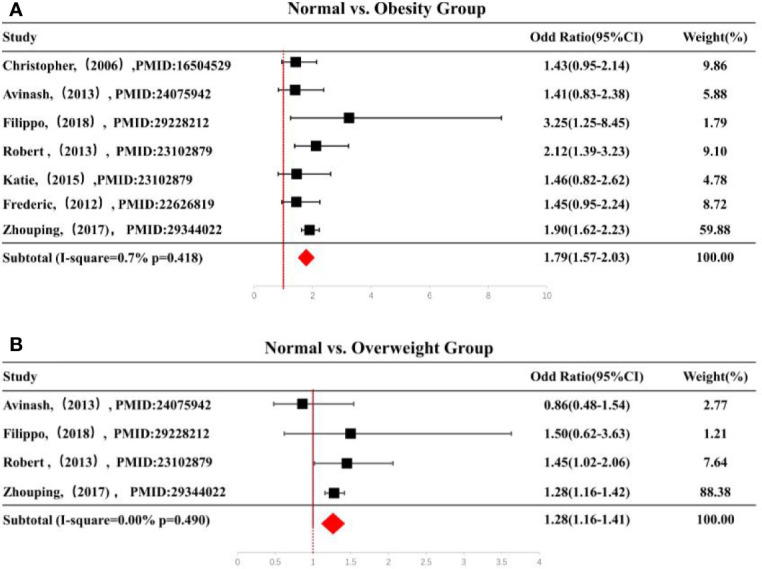
**(A)** Forest plot of ORs of obesity vs. normal weight and **(B)** forest plot of ORs of overweight vs. normal weight. Black squares indicate the OR in each study, horizontal lines represent the 95% CI. OR, odd ratio; CI, confidence interval; BMI, body mass index.

Compared with normal weight, overweight group with cardiac surgery had higher AKI risk. A similar association was found in obese subgroup (OR: 1.79, 95% CI: 1.57–2.03) (**Figure 2A**).The combined ORs (95% CI) were 1.28 (1.16–1.41) (**Figure 2B**). The heterogeneity among the studies were low (*I*^2^ = 0.0% for overweight, *I*^2^ = 0.7% for obese groups) (**Supplementary Table 2**).

When stratified by Geographic area, as shown in **Supplementary Table 2**, there was a higher AKI risk in UK and China for overweight and obese groups in relation to the normal weight group. In the stratified analysis by diagnosis standard, the overweight subjects diagnosed using the KDIGO standard suffered from increased AKI risk compared to those with normal weight. When stratified by Geographic area, as shown in **Supplementary Table 2**, there was a higher AKI risk in UK and China for overweight and obesity groups compared with normal weight individuals. In the stratified analysis by diagnosis standard, the subjects with KDIGO diagnosis standard suffered from increased AKI risk for overweight compared with normal weight. In addition, age, hypertension, diabetes and smoking were also significantly related to AKI risk for overweight and obesity subgroup compared with normal weight. Both the funnel plot and Egger’s test showed no publication bias (**Supplementary Figure 2**).

## Discussion

In this cohort study, we recruited 1,601 individuals post-cardiac surgery. We found overweightness to be an independent risk factor of CS-AKI. The outcome of the meta-analysis further confirmed the correlation between overweightness and obesity and increased incidence of CS-AKI. Similar results were observed in the subgroup analysis by geographic area and AKI diagnostic standard.

In line with our results, previous studies reported BMI as an independent risk factor of CS-AKI. For instance, Ko and colleagues demonstrated that high BMI was an independent risk factor of AKI (27). Also, there was no significant correlation between BMI and renal insufficiency in elderly patients undergoing coronary artery bypass grafting from the findings by Reis et al. (28). Roh and colleagues reported that age and BMI were not independent risk factors for CS-AKI (29). Another study reported that extreme obesity (BMI > 40 kg/m^2^) was associated with AKI, rather than obesity (BMI, 30–40 kg/m^2^) (24). These divergent conclusions perhaps stemmed from differences in AKI diagnostic criteria, differences in geographical regions of study, dissimilarities in patient inclusion criteria, and different adjustment for identified AKI risk factors.

With the increasing incidence of CS-AKI (30, 31), the role of abnormal BMI in AKI has drawn more and more attention in the world, but the deeply mechanism is still unclear. Obesity can significantly alter renal hemodynamics, which may explain the increased susceptibility of obese patients to AKI (32). Increased renal plasma flow and glomerular filtration rate due to hemodynamic changes may lead to higher filtration rate or high filtration syndrome, making the kidneys prone to damage (33). In addition, obese patients often suffer from cor-pulmonale due to insufficient ventilation, sleep apnea and pulmonary hypertension leading to sodium dependence and peripheral venous congestion (34, 35). It also has been reported that peripheral venous congestion in turn could result in increased renal venous pressure, thereby reducing urine formation (36). Another factor contributing to the effect of obesity on AKI is the challenge of correctly assessing the status of vascular content and adequate fluid therapy. Obesity shows influence on the dosage of potential nephrotoxic drugs *via* affecting many pharmacokinetic factors (37).

Of note, obese ICU patients have an increased risk of elevated intra-abdominal pressure, which could lead to renal insufficiency due to venous congestion and poor perfusion of arterial organs (38, 39). In addition, obesity-related cardiac changes such as increased left ventricular hypertrophy and direct myocardial infiltration may also alter renal perfusion (40).

The term “obesity paradox” has been used to describe similar or improved survival rates in critically-ill obese patients. One explanation for this phenomenon is that obese patients benefit from metabolic or nutritional reserves, which ultimately leads to increased survival under diseased conditions (41). A multicenter observational study of 940 patients found no link between obesity (BMI > 30 kg/m^2^) and increased risk of major perioperative complications during trans-catheter aortic valve implementation. However, obese patients had a greater incidence of CS-AKI (stage I). In this study, a clear association between increased operative mortality and increased BMI was found, thereby refuting the “obesity paradox” for operative outcomes. Conflicting findings of previous studies could be due to differences in patient cohort, risk stratification and BMI classification (42–44). In a contemporary multi-agency regional cohort study of 13,637 patients, researchers showed that increased BMI significantly elevated the likelihood of major post-operative morbidities, especially acute renal failure and pneumonia (45).

Metabolic syndrome (MetS) has been reported as a risk factor for postoperative kidney injury after off-pump coronary artery bypass surgery (OPCAB) (46). But our study show that there is no significant difference in the effect of diabetes (p = 0.152) and hyperlipemia (p = 0.099) on CS-AKI, small sample size may be relevant with this conclusion. Conclusively, Future work with larger sample size, especially the diabetes and hyperlipemia patients are needed. The relationship between hyperlipemia and CS-AKI still needs to be studied in future studies with a larger multi-center sample. Previous report revealed that of mean arterial pressure (MAP) is a risk factor for the progression of AKI (47). Continued high blood pressure produces increased vascular hydrostatic pressure with consequent thickening of arterial wall. The alterations make the kidney more prone to develop pre-renal AKI in case of hypo-perfusion (48). Consistent with this view, our study confirmed that patients with hypertension medication history had higher risk of CS-AKI than their counterparts.

The limitations of this study are herein outlined: First, although the surgery principles governing conduct with respect to patient are the same, there are still differences in surgical operation on the details. It’s hard to eliminate the impact of surgical procedures on the incidence of AKI. Second, samples undergoing cardiac surgery were recruited from a single center, which perhaps introduced bias to the result.

In conclusion, our findings are consistent with the generally held consensus that abnormal BMI is an independent risk factor for the development of CS-AKI. Patients whose BMI were below 18.5 or above 28.0 had a higher incidence of CS-AKI in our cohort study. BMIs > 18.5 were independent predictors of CS-AKI.

## Data Availability Statement

All datasets generated for this study are included in the article/**Supplementary Material**.

## Ethics Statement

All experiment involved human are approved by the Ethics Committee of Jiangsu Province Hospital. All participants agreed to participate this study and signed informed consent document at the time of enrollment. This study complied with the Helsinki Declaration.

## Author Contributions

SJ, PX, S-PM, NS, and KL conceived the study. NS, KL, HW, ML, HM, and XX recruited the CS-AKI patients and collected clinical data. NS, XL, and LY reviewed eligible publications and extracted the data needed for the meta-analysis. NS, YF, and SZ performed statistical analysis. All authors contributed to the article and approved the submitted version.

## Funding

This project funded by Jiangsu Provincial Science Foundation (Grant No. BK20190555) and the National Natural Science Foundation of China (Grant No. 81900780).

## Conflict of Interest

The authors declare that the research was conducted in the absence of any commercial or financial relationships that could be construed as a potential conflict of interest.
