# Supplementary material for: The Association Between Obesity and Risk of Acute Kidney Injury After Cardiac Surgery
Source: Front Endocrinol (Lausanne). 2020 Oct 6;11:534294. doi: 10.3389/fendo.2020.534294 (PMC7573233; doi:10.3389/fendo.2020.534294)
Supplement: Supplementary file 1 [file DataSheet_1.docx]

**Supplementary Figure 1**

**
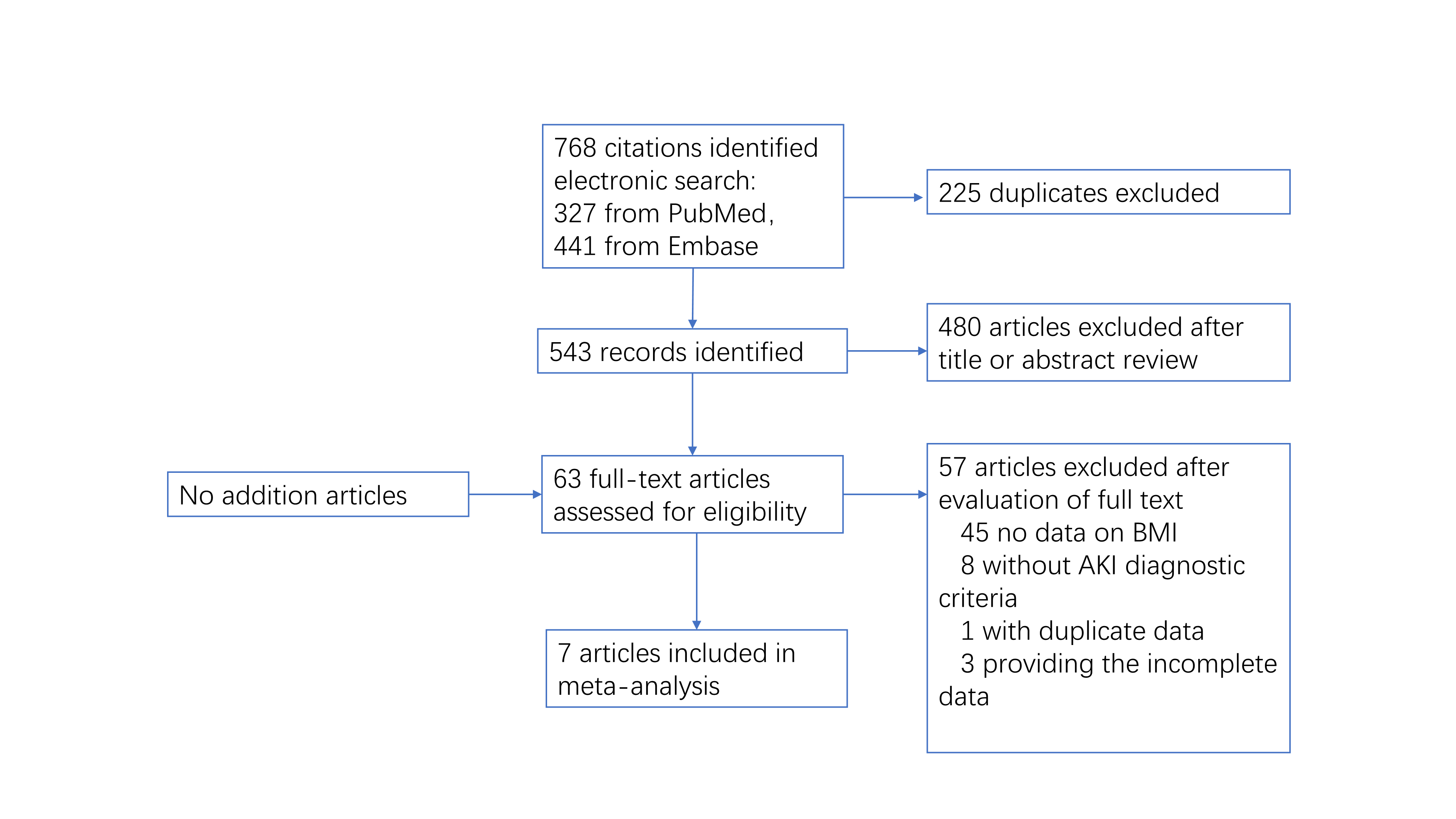
**

Supplementary figure 1. Flowchart of articles selection with criteria.

**Supplementary Figure 2.**


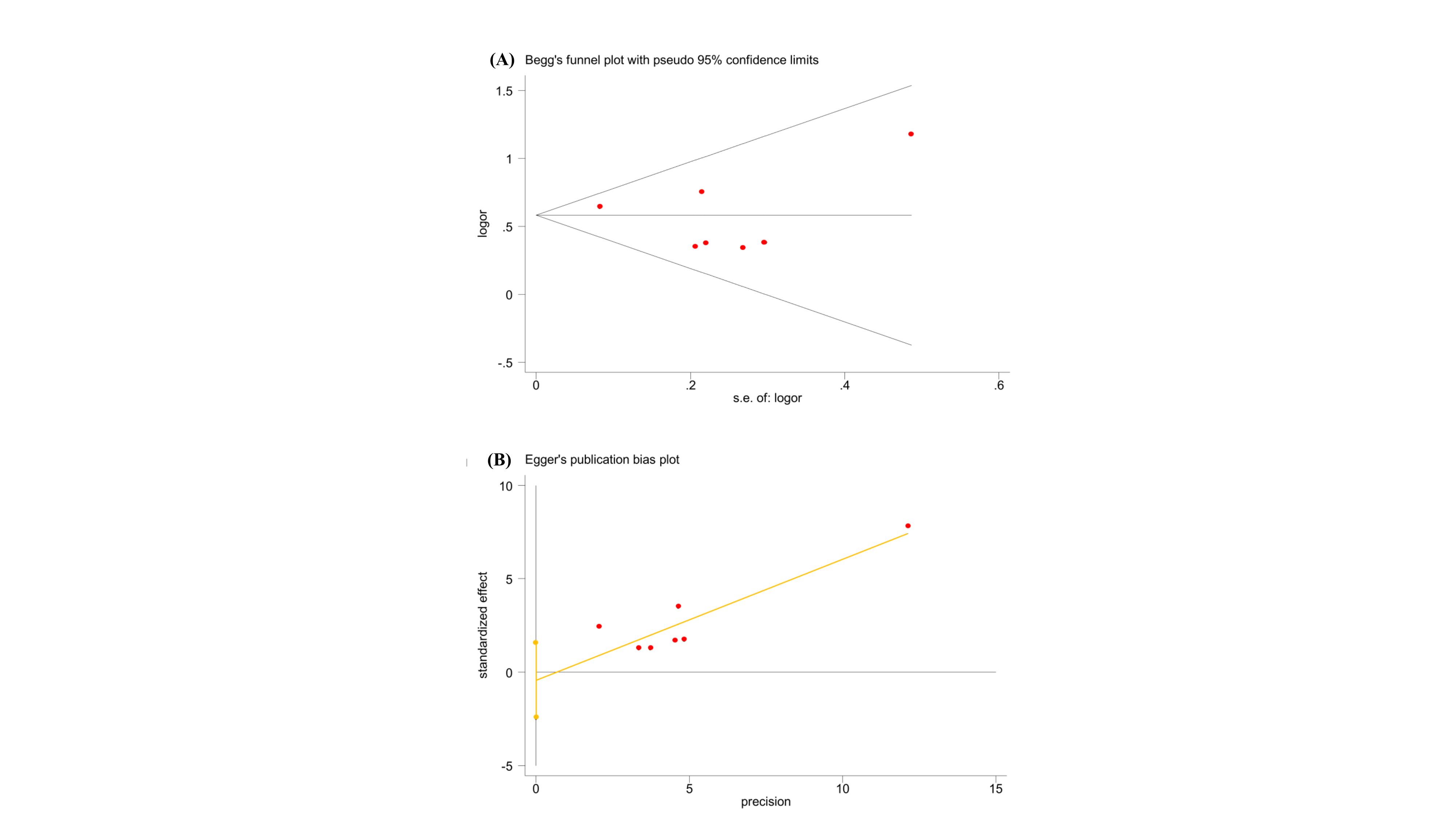


Supplementary Figure 2. **(a)** Funnel plot of abnormal BMI and CS-AKI **(b)** Egger’s Publication bias plot
