# Supplementary material for: The Association Between Obesity and Risk of Acute Kidney Injury After Cardiac Surgery
Source: Front Endocrinol (Lausanne). 2020 Oct 6;11:534294. doi: 10.3389/fendo.2020.534294 (PMC7573233; doi:10.3389/fendo.2020.534294)
Supplement: Supplementary file 2 [file Table_1.docx]

**Supplementary table 1**. Characteristics of published studies on BMI and risk of CS-AKI.

| **First author, year, country** | **Study period** | **Study size, sex, number of cases** | **AKI diagnostic criteria** | **BMI** | **OR (95%CI)** | **Adjustment factors** |
| --- | --- | --- | --- | --- | --- | --- |
| Christopher, (2006), USA | 1999-2004 | 1867, (M, 1239), 100 | RIFLE | 20-30 | 1.00 | Age, sex, diabetes, hypertension |
|  |  |  |  | 30-40 | 1.22 (0.77-1.93) |  |
|  |  |  |  | ≥40 | 2.08 (1.24-3.44) |  |
| Avinash, (2013), USA | 2006-2010 | 376, (M, 260), 147 | AKIN | <25 | 1.00 | Age, diabetes mellitus, hypertension, preexisting kidney disease, type of surgery, PRBC transfusions, baseline creatinine median, euroscore median, CPB time median |
|  |  |  |  | 25-30 | 0.62（0.32-1.22） |  |
|  |  |  |  | 30-35 | 0.92 (1.45-1.89) |  |
|  |  |  |  | 35-40 | 0.53 (0.21-1.31) |  |
|  |  |  |  | ≥40 | 2.39 (0.98-5.82) |  |
| Filippo, (2018), UK | 2000-2015 | 715, (M, 478), 32 | RIFLE | <20 | 0.99 (0.21-4.68) | Sex, hypertension, smoking history, NYHA class III–IV, CPB time |
|  |  |  |  | 20–24.99 | 1.00 |  |
|  |  |  |  | 25–29.99 | 0.73 (0.30-1.79) |  |
|  |  |  |  | >30 | 0.73 (0.22-2.43) |  |
| Robert, (2013), Europe |  | 940, (M, 506), 216 | AKIN | <18.5 | 1.17 (0.39-3.52) | Age, sex, diabetes mellitus, BMI, glomerular filtration rate, aortic valve area |
|  |  |  |  | 18.5-24.9 | 1.00 |  |
|  |  |  |  | 25-29.9 | 0.69 (0.49-0.98) |  |
|  |  |  |  | >30 | 0.47 (0.31-0.72) |  |
| Katie, (2015), UK | 2009-2010 | 432, (M, 338), 57 | Unknown | <30 | 1.00 | Age, diabetes mellitus, obesity, CPB, recent MI, postoperative vasopressor used, |
|  |  |  |  | ≥30 | 2.12 (1.27-3.54) |  |
| Frederic, (2012), USA | 2005-2012 | 445, (M, 296), 112 | AKIN | <30 | 1.00 | BMI, systolic BP, central venous pressure |
|  |  |  |  | ≥30 | 1.29 (1.06-1.56) |  |
| Zhou ping, (2017), China | 2009-2014 | 8455, (M, 4706), 2855 | KDIGO | <18.5 | 1.18 (0.96-1.46) | Age, sex, diabetes mellitus, diabetes mellitus, BMI, chronic heart failure, preoperative coronary angiography, album baseline, blood urea nitrogen, SCr baseline, uric baseline, valve surgery |
|  |  |  |  | 18.5-24 | 1.00 |  |
|  |  |  |  | 24-28.0 | 1.20 (1.04-1.37) |  |
|  |  |  |  | ≥30 | 1.50 (1.20-1.88) |  |

BMI, body mass index; AKI, acute kidney injury; SCr, serum creatinine values; RIFLE standard was formulated by ADQI (Acute Dialysis Quality Initiative) working group in 2004; AKIN was revised by Acute Kidney Injury Network in 2007; KDIGO: The Global Organization for the Improvement of Prognosis of Kidney Diseases (KDIGO) proposed a comprehensive and unified AKI diagnostic and grading criteria in the 2012 AKI Clinical Practice Guidelines; OR, odd ratio; CI confidence interval; PRBC, packed red blood cells; CPB time, cardiopulmonary bypass time; NYHA, New York Heart Association (classification); MI, myocardial infarction; BP, blood pressure.

**Supplementary Table 2**. Subgroup analysis of BMI and CS-AKI risk in the meta-analysis.

|  | **Overweight** | |  |  | |  | **Obesity** | |  |  |  |
| --- | --- | --- | --- | --- | --- | --- | --- | --- | --- | --- | --- |
|  | ***n*** | **OR (95%CI)** | **Heterogeneity** | | |  | ***n*** | **OR (95%CI)** | **Heterogeneity** | | |
|  |  |  | ***Q*** | ***p*** | ***I*^2^%** |  |  |  | ***Q*** | ***p*** | ***I*^2^%** |
| **All study** | 4 | 1.28 (1.16-1.41) | 3.09 | 0.378 | 0.0 |  | 7 | 1.79 (1.57-2.03) | 6.05 | 0.418 | 0.7 |
| **Geographic area** |  |  |  |  |  |  |  |  |  |  |  |
| **America** | 1 | 0.86 (0.48-1.54) |  |  |  |  | 3 | 1.42 (1.08-1.89) | 0.01 | 0.995 | 0.0 |
| **UK** | 2 | 1.46 (1.05-2.02) | 0.01 | 0.944 | 0.0 |  | 2 | 2.27 (1.55-3.34) | 0.65 | 0.42 | 0.0 |
| **Europe** | 0 |  |  |  |  |  | 1 | 1.46 (0.95-2.24) |  |  |  |
| **China** | 1 | 1.28 (1.16-1.41) |  |  |  |  | 1 | 1.90 (1.62-2.24) |  |  |  |
| **AKI** **diagnosis standard** |  |  |  |  |  |  |  |  |  |  |  |
| **RIFLE** | 1 | 1.50 (0.62-3.63) |  |  |  |  | 2 | 1.91 (0.88-4.16) | 2.43 | 0.119 | 58.9 |
| **AKIN** | 2 | 1.18 (0.71-1.95) | 2.29 | 0.131 | 56.2 |  | 3 | 1.71 (1.29-2.28) | 1.80 | 0.406 | 0.0 |
| **KDIGO** | 1 | 1.28 (1.16-1.42) |  |  |  |  | 1 | 1.46 (0.95-2.24) |  |  |  |
| **Unknown** | 0 |  |  |  |  |  | 1 | 1.90 (1.62-2.24) |  |  |  |
| **Adjustment factors** |  |  |  |  |  |  |  | 1.46 (0.95-2.24) |  |  |  |
| **Age** |  |  |  |  |  |  |  |  |  |  |  |
| **Yes** | 2 | 1.30 (1.16-1.41) | 0.45 | 0.503 | 0.0 |  | 3 | 1.85 (1.59-2.16) | 2.09 | 0.351 | 4.5 |
| **No** | 2 | 1.03 (0.62-1.70) | 1.08 | 0.300 | 7.0 |  | 4 | 1.53 (1.17-2.04) | 2.57 | 0.462 | 0.0 |
| **Hypertension** |  |  |  |  |  |  |  |  |  |  |  |
| **Yes** | 3 | 1.27 (1.15-1.40) | 1.90 | 0.338 | 0.0 |  | 4 | 1.76 (1.41-2.19) | 4.03 | 0.258 | 25.6 |
| **No** | 1 | 1.45 (1.02-2.06) |  |  |  |  | 3 | 1.69 (1.30-2.21) | 1.81 | 0.405 | 0.0 |
| **Hyperlipemia** |  |  |  |  |  |  |  |  |  |  |  |
| **Yes** | 0 |  |  |  |  |  | 1 | 1.43 (0.95-2.14) |  |  |  |
| **No** | 4 | 1.28 (1.16-1.41) | 2.42 | 0.490 | 0.0 |  | 6 | 1.79 (1.57-2.03) | 4.71 | 0.452 | 0.0 |
| **Diabetes** |  |  |  |  |  |  |  |  |  |  |  |
| **Yes** | 2 | 1.30 (1.17-1.43) | 0.45 | 0.503 | 0.0 |  | 4 | 1.80 (1.55-2.09) | 3.22 | 0.359 | 6.8 |
| **No** | 2 | 1.03 (0.62-1.70) | 1.08 | 0.300 | 7.0 |  | 3 | 1.64 (1.09-2.47) | 2.45 | 0.294 | 18.4 |
| **Smoking** |  |  |  |  |  |  |  |  |  |  |  |
| **Yes** | 1 | 1.50 (0.62-3.63) |  |  |  |  | 1 | 3.25 (1.25-8.45) |  |  |  |
| **No** | 3 | 1.28 (1.11-1.47) | 2.29 | 0.318 | 12.8 |  | 6 | 1.77 (1.56-2.01) | 4.51 | 0.479 | 0.0 |

BMI, body mass index; AKI, acute kidney injury; SCr, serum creatinine values; RIFLE standard was formulated by ADQI (Acute Dialysis Quality

Initiative) working group in 2004; AKIN was revised by Acute Kidney Injury Network in 2007; KDIGO: The Global Organization for the Improvement

of Prognosis of Kidney Diseases (KDIGO) proposed a comprehensive and unified AKI diagnostic and grading criteria in the 2012 AKI Clinical Practice Guidelines; OR, odd ratio; CI confidence interval; PRBC, packed red blood cells; CPB time, cardiopulmonary bypass time; NYHA, New York Heart

Association (classification); MI, myocardial infarction; BP, blood pressure
